# Plasma skimming efficiency of human blood in the spiral groove bearing of a centrifugal blood pump

**DOI:** 10.1007/s10047-020-01221-9

**Published:** 2020-10-28

**Authors:** Daisuke Sakota, Kazuki Kondo, Ryo Kosaka, Masahiro Nishida, Osamu Maruyama

**Affiliations:** Artificial Organ Research Group, Health and Medical Research Institute, National Institute of Advanced Industrial Science and Technology (AIST), 1-2-1 Namiki, Tsukuba, Ibaraki 305-8564 Japan

**Keywords:** Plasma skimming, Hydrodynamic bearing, Centrifugal blood pump, Hemolysis, Optics of blood

## Abstract

This work investigates the plasma skimming effect in a spiral groove bearing within a hydrodynamically levitated centrifugal blood pump when working with human blood having a hematocrit value from 0 to 40%. The present study assessed the evaluation based on a method that clarified the limitations associated with such assessments. Human blood was circulated in a closed-loop circuit via a pump operating at 4000 rpm at a flow rate of 5 L/min. Red blood cells flowing through a ridge area of the bearing were directly observed using a high-speed microscope. The hematocrit value in the ridge area was calculated using the mean corpuscular volume, the bearing gap, the cross-sectional area of a red blood cell, and the occupancy of red blood cells. The latter value was obtained from photographic images by dividing the number of pixels showing red blood cells in the evaluation area by the total number of pixels in this area. The plasma skimming efficiency was calculated as the extent to which the hematocrit of the working blood was reduced in the ridge area. For the hematocrit in the circuit from 0 to 40%, the plasma skimming efficiency was approximately 90%, meaning that the hematocrit in the ridge area became 10% as compared to that in the circuit. For a hematocrit of 20% and over, red blood cells almost completely occupied the ridge. Thus, a valid assessment of plasma skimming was only possible when the hematocrit was less than 20%.

**Electronic supplementary material:**

The online version of this article (10.1007/s10047-020-01221-9) contains supplementary material, which is available to authorized users.

## Introduction

Understanding the blood flow dynamics in mechanical circulatory support devices is important for the development of more hemocompatible devices, with the aim of preventing thrombosis and hemolysis. Consequently, many researchers have investigated and simulated such dynamics using computational fluid dynamics (CFD) and particle image velocimetry [1–5]. However, these conventional studies have assessed blood flow solely on the macroscopic level. In fact, it is challenging to precisely simulate or even to directly visualize localized bearing areas in a rotary blood pump, which are likely the primary regions associated with the destruction of blood cells. As such, it would be helpful to have a better understanding of blood flow dynamics on the scale of individual cells at such hydrodynamic bearing regions, to assist in the development of novel hemocompatible devices.

The phenomenon of plasma skimming (PS) is observed in microvessels. This effect is defined as a reduction in the hematocrit (HCT) value in a branched vessel as compared to that in a main vessel due to the lower migration of red blood cells (RBCs) into the branch. PS was first described by Krogh [6] and has typically been investigated by observations during in vivo microcirculation or with in vitro microfluidic devices [7–11], although some researchers have also utilized artificial organs. In 1985, Lewandowski and Nose et al. reported that a PS layer is formed at membrane surfaces and speculated that controlling this effect may assist in the further development of hemofiltration [12]. Also, in 2004, Kink and Reul applied a spiral groove bearing (SGB) to an axial flow blood pump for the first time and developed a system in which the entry of RBCs into the bearing gap is inhibited based on the PS effect, such that hemolysis does not occur [13]. Several other studies of the PS effect in conjunction with hydrodynamic bearings have been performed but did not involve the direct observation and quantification of the degree of this phenomenon [14–16]. Although concepts to either utilize or control the PS effect have been established in past research, few quantitative evaluations have been performed. In 2016, our own group reported a method for the quantitative assessment of the PS effect in a hydrodynamically levitated centrifugal blood pump [17]. This work involved the first-ever direct quantitative analysis of PS in an SGB, using bovine blood at an HCT of 1%.

Herein, we demonstrate the quantitative assessment of the PS effect in an SGB, working with human blood, whose the HCT range from 0 to 40%. The maximum limitation of the evaluation of PS effect for HCT is clarified. Both the evaluation method and the theoretical basis of PS are examined and the limitations of this technique are discussed.

## Materials and methods

### Ethical approval

Human whole blood used in the study was donated by the Japanese Red Cross Society. The study was approved by the Life Science Experiment Application Committee at the National Institute of Advanced Industrial Science and Technology (Approved Number: hi 2017-225), Japan.

### Experimental setup

**Fig. 1:**
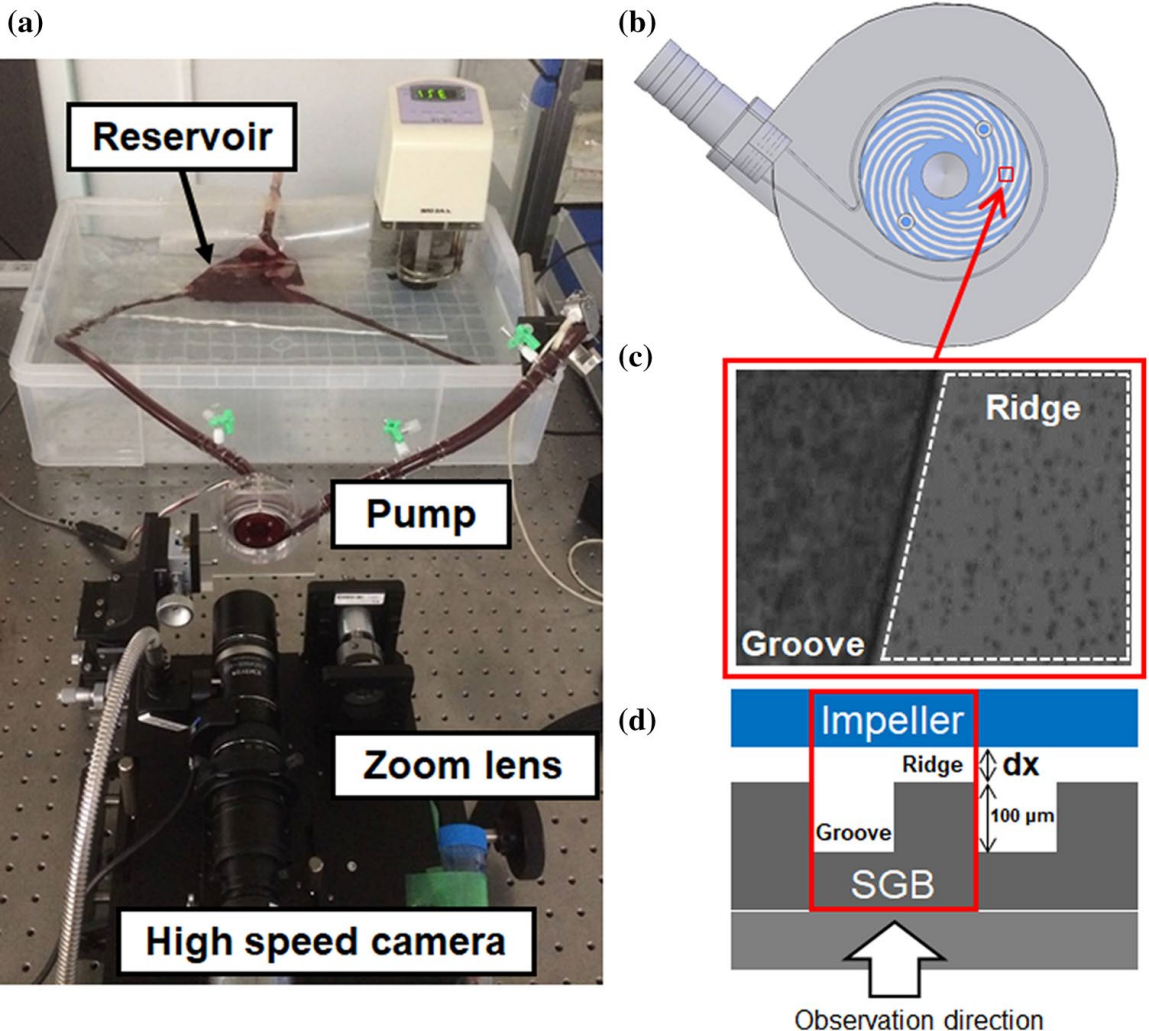
Experimental setup. **a** Photographic image of the plasma skimming evaluation system. **b** Diagram showing the observation point at a single groove and ridge in the spiral groove bearing of the hydrodynamically levitated centrifugal blood pump. **c** Microscopic image obtained using a high-speed camera. The ridge area surrounded by the dotted line is the region in which plasma skimming was evaluated. **d** Cross-sectional view of the observation point. Here, dx is the bottom bearing gap

The PS evaluation system was constructed as described previously [17], and is pictured in Fig. 1a. This apparatus incorporated a hydrodynamically levitated centrifugal blood pump developed by our group, the structure of which has been reported in detail in a prior publication [18]. The rotational speed of the pump was maintained at 4000 rpm throughout the experiments, with a flow rate of 5 L/min. The flow circuit comprised a reservoir, polyvinyl chloride tubing with an inner diameter of 3/8 in., a sampling port, an adjustable resistor and the blood pump. The blood temperature was maintained at 37 °C. The pump components were made of transparent polymethyl methacrylate. The closed-type impeller had six vanes, a diameter of 37 mm, and a height of 26 mm, and was levitated by hydrodynamic bearings acting in the radial and thrust directions. SGBs were attached to the top and bottom casing to suspend the impeller in the thrust direction, and the bottom part between the impeller and the SGB was monitored using a high-speed camera (VW-9000; Keyence Corp., Osaka, Japan) with a long-distance high-performance zoom lens (VH-Z50W; Keyence Corp.). The shutter speed and frame rate were set to 1/900,000 s and 15,000 frames/s respectively, and a xenon lamp (MAX-303; Asahi Spectra Corp., Tokyo, Japan) was attached to the zoom lens as a light source. The camera was set up so that a single groove and ridge could be observed, as shown in Fig. 1b, and an actual image obtained from the high-speed camera is shown in Fig. 1c. Figure 1d presents a cross-sectional view of the observation point. The ridge area at this point represents the region at which the highest shear rate appeared in the pump. The shear rate was estimated to be approximately 10^5^ 1/s or higher at 4000 rpm. The region labeled “dx” in this figure represents the bottom bearing gap or the levitation distance, which varied with the hydrodynamic force generated by the SGB.

In the present experiments, the PS effect was investigated over the range of HCT values from approximately 0–40% by adding phosphate buffered saline (PBS) to the circuit. At each HCT, the ridge area was imaged by the camera over three rotational phases of the impeller and each trial was repeated five times.

### Plasma skimming efficiency

In this study, the PS efficiency (PSE) was defined as follows:

1 $${\text{PSE}} = 1 - \frac{{{\text{HCT}}_{{\text{R}}} }}{{{\text{HCT}}_{{\text{W}}} }},$$

where HCT_W_ is the hematocrit value of working or circulating blood in the circuit and HCT_R_ is the hematocrit in the ridge area under observation. At a PSE value of 100%, HCT_R_ will be 0%, meaning that all RBCs escape from the highest shear-stress area in the ridge to the lower shear-stress area in the groove. HCT_W_ values were determined based on the blood aliquots taken from the sampling port located in the circuit tube. The calculation of HCT_R_ has been described in a previous paper [17], and is based on assessing the optical scattering coefficient of the blood. This coefficient is calculated assuming that the RBCs are uniformly spread throughout the ridge, using the following equation [19–24]:

2 $$\mu_{{\text{s}}} = \frac{{{\text{HCT}}_{{\text{R}}} }}{{{\text{MCV}}}}\sigma ,$$

where *µ*_s_ is the scattering coefficient (with units of 1/µm), MCV is the mean corpuscular volume of the RBCs and σ is the scattering cross-section. Equation (2) indicates that a photon travelling through blood will definitely hit an RBC within a distance of 1/*µ*_s_. As such, if the bottom bearing gap is dx = 1/*µ*_s_ at HCT_R_, the two-dimensional ridge area shown in Fig. 1c will be occupied by RBCs. Therefore, we can write the following relation:

3 $$\mu_{{\text{s}}} = Q/dx,$$

where *Q* is the occupancy of RBCs in the ridge area. The value of *Q* is obtained by dividing the number of pixels containing RBCs in the evaluation area by the total number of pixels in this region [17]. Based on Eqs. (2) and (3), HCT_R_ can be calculated as follows:

4 $${\text{HCT}}_{{\text{R}}} = \frac{{Q \times {\text{MCV}}}}{dx \times \sigma }.$$

The MCV can be determined using an automated blood cell analyzer (Celltac α MEK-6450, Nihon Kohden Corp., Tokyo, Japan). Sakota and Takatani showed that σ can be regarded as equivalent to the geometrical cross-section of a biconcave model RBC when working with visible or near-infrared light [24], and can be calculated as follows:

5 $$\sigma = \pi \left( {\frac{{3{\text{MCV}}}}{4\pi C}} \right)^{2/3} .$$

where *C* (= 0.39023) is the ratio of the volume of a biconcave RBC to that of a sphere having a radius equal to the long-axis radius of the RBC.

### Relationship between bearing gap size and viscosity

**Fig. 2:**
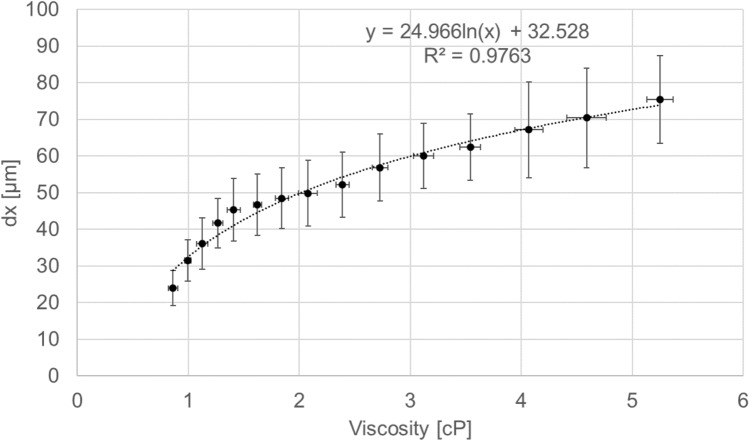
Relationship between the bottom bearing gap, dx, and the viscosity of the working fluid. Each data point represents the average of three replicate experiments

In this work, HCT_R_ values were calculated using Eq. (4). During the various trials, dx changed along with the hydrodynamic force, which was dependent on the viscosity of the blood. The viscosity, in turn, was primarily determined by the HCT value. In a previous study [17], dx could be measured using a laser displacement meter because the HCT value was quite low (1%), but this becomes more difficult at higher HCT values. Thus, in the work reported herein, we determined the relationship between dx and viscosity separately in additional experimental trials that did not use blood. In these experiments, the circuit was made of the same materials and the pump was also operated at 5 L/min and 4000 rpm, but a glycerol (Wako Pure Chemical Industries Ltd., Osaka, Japan)/water solution was used as the working fluid. The viscosity of this fluid could be modified by varying the proportion of glycerol and was ascertained with a viscometer (DVM-E-II, Tokimec Inc., Tokyo, Japan). The dx values were measured using a laser confocal displacement meter with a resolution of 0.2 µm (LT8110; Keyence Corp.) and each experiment was conducted three times, with the results as shown in Fig. 2. From these data, it is evident that dx increased as the viscosity was increased. Based on these results, dx values in the experiments using blood were estimated from the viscosity of the blood at each HCT_W_ as measured with a viscometer throughout each trial.

### Evaluation of the reliability of plasma skimming efficiency values

When calculating HCT_R_, the dx at each HCT_W_ was obtained by substituting the measured viscosity into the dx-viscosity correlation provided in Fig. 2. However, paradoxically, the viscosity of the blood in the ridge area should equal the viscosity at the HCT_R_, and so another index, PSE_min_(dx = 25 µm) was also calculated. If PS perfectly occurs, the HCT_R_ should be close to zero and the viscosity becomes plasma. Then, since the viscosity of plasma is close to that of water, based on this assumption and Fig. 2, dx will have a minimum value of approximately 25 µm at the viscosity (0.86 cP) of pure water at 37 °C. Based on Eq. 4, the resulting HCT_R_ value will be overestimated when dx is given a constant minimum value of 25 µm. Therefore, the use of PSE_min_(dx = 25 µm) provides the most conservative estimate of PSE.

It is important to understand the relationship between *Q* and dx when evaluating the PSE at high HCT values. In the case of *Q* = 1, the RBCs occupy only the ridge area, such that the RBC layer will consist of just one RBC that covers the ridge. If *Q* = 2, the layer will consist of two RBCs. However, the present system can only evaluate *Q* over the range of 0 ≤ *Q* ≤ 1 because of the two-dimensional imaging that is employed, as shown in Fig. 1c. The range over which the PSE can be examined can be obtained from the following equation:

6 $${\text{PSE}}_{{\text{L}}} = 1 - \frac{1}{{Q_{{\text{W}}} }},$$

where

7 $$Q_{{\text{W}}} = \frac{{{\text{HCT}}_{{\text{W}}} \times dx \times \sigma }}{{{\text{MCV}}}}.$$

*Q*_W_ is calculated by substituting HCT_W_ for HCT_R_ in Eq. (4). Consequently, PSE can be evaluated when PSE_L_ ≤ PSE ≤ 1. It should also be noted that, in the case that Q_W_ < 1, Eq. (6) indicates that PSE_L_ will have a value of 0.

## Results

**Fig. 3:**
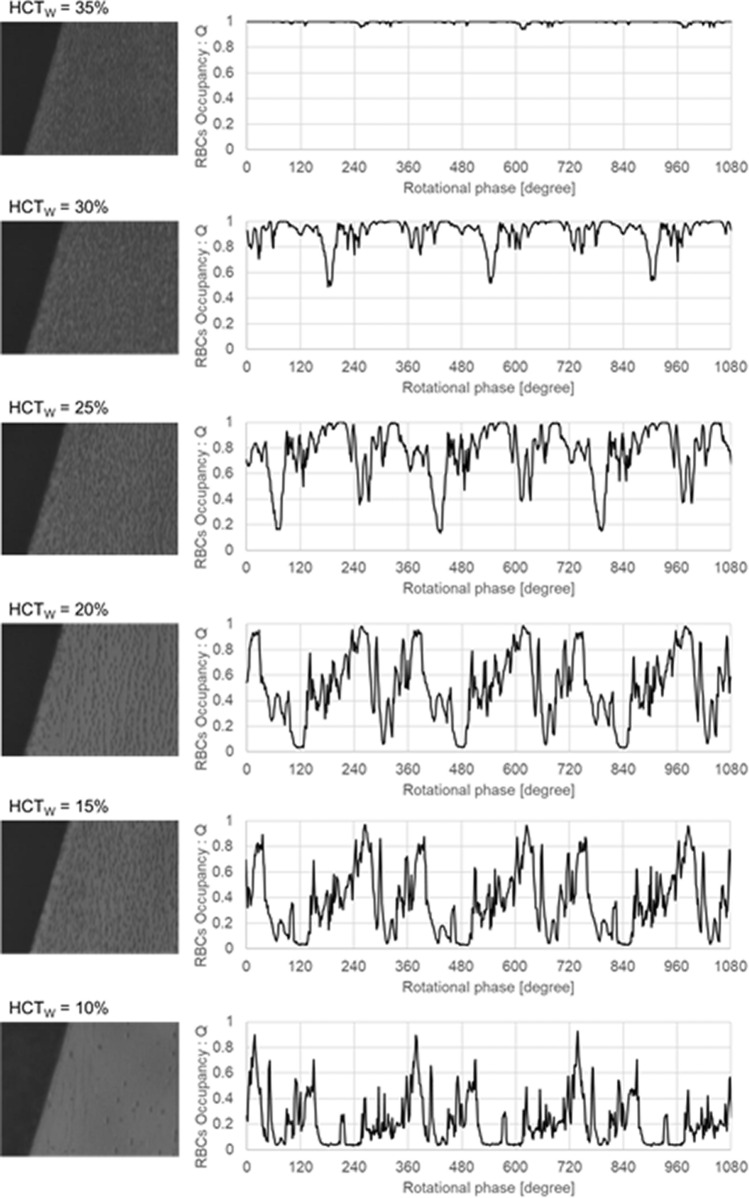
Typical high-speed photographic images of the observation point at various HCT_W_ and the variations in Q with respect to the impeller rotational phase

**Fig. 4:**
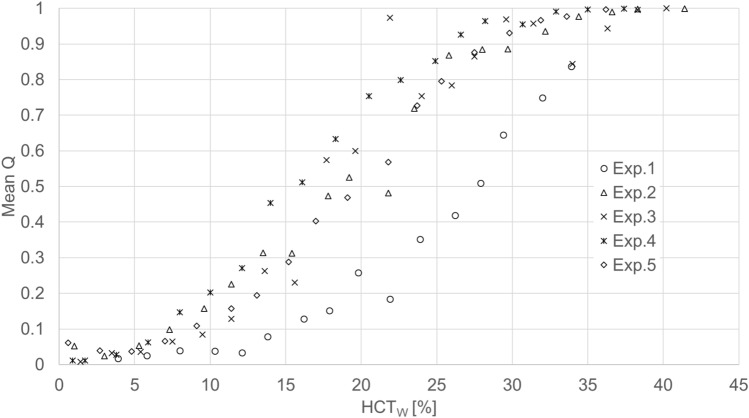
Relationship between the mean *Q* value obtained by averaging *Q* shown in this figure over three rotational phases of the impeller and HCT_W_

**Fig. 5:**
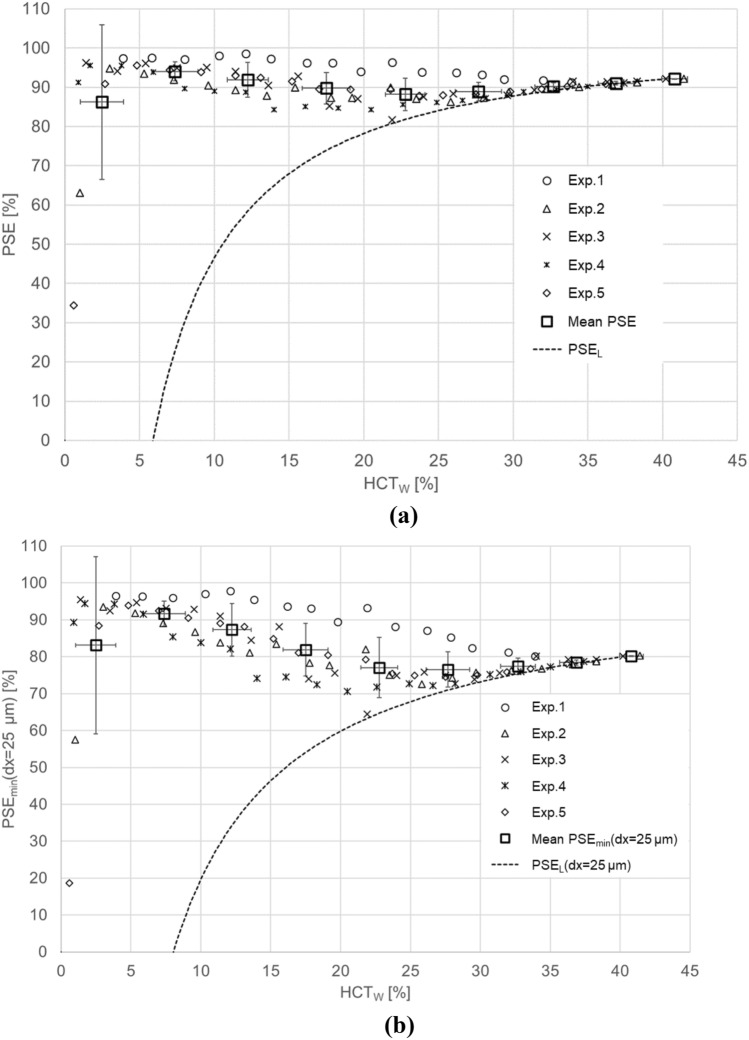
**a** Plasma skimming efficiency, PSE, and the evaluation limit, PSE_L_, as functions of HCT_W_. **b** Most conservative estimated plasma skimming efficiency, PSE_min_(dx = 25 µm), and evaluation limit, PSE_L_(dx = 25 µm), as functions of HCT_W_

The mean (± standard deviation) MCV obtained from five replicate trials was 94 ± 2.7. The estimated dx values in the HCT_W_ whose viscosity in each blood was measured range from 0 to 40% were approximately 25–60 µm. The relationship between dx and HCT_W_ was linear. Figure 3 provides typical high-speed photographic images acquired at HCT_W_ values from 10 to 35%, from which it is evident that the number of RBCs in the ridge area was decreased as HCT_W_ was decreased. The plots in Fig. 3 indicate significant variations in *Q* with respect to the rotational phase for various HCT_W_, so mean values of *Q* were used for the evaluation of PSE. Mean *Q* was obtained by averaging *Q* shown in Fig. 3 over three rotational phases of the impeller. Figure 4 shows the relationship between the mean *Q* values and HCT_W_. By using Fig. 4, PSE and PSE_min_(dx = 25 µm) were calculated and the results are shown in Fig. 5a, b, respectively. The mean values for PSE and PSE_min_(dx = 25 µm) with respect to HCT_W_ are summarized in Table 1. Thus, for HCT_W_ > 20%, PSE and PSE_min_(dx = 25 µm) both approached PSE_L_.

**Table 1:** Mean value (± standard deviation) for PSE and PSE_min_(dx = 25 µm) with respect to HCT_W_

HCT_w_ (%)	PSE (%)	PSE_min_ (dx = 25 µm) (%)
2.5 ± 1.4	86.3 ± 19.7	83.1 ± 24.0
7.4 ± 1.5	94.1 ± 2.4	91.7 ± 3.4
12.2 ± 1.4	91.9 ± 4.5	87.3 ± 7.1
17.5 ± 1.6	89.8 ± 3.9	81.9 ± 7.1
22.8 ± 1.3	88.2 ± 4.2	77.1 ± 8.2
27.7 ± 1.5	88.9 ± 2.4	76.5 ± 4.8
32.7 ± 1.2	90.2 ± 1.0	77.4 ± 2.2
36.9 ± 1.2	91.0 ± 0.5	78.5 ± 0.7
40.8 ± 0.8	92.0 ± 0.0	80.2 ± 0.1

The number of experiments is five times

## Discussion

The developed system and the experimental method based on the optical theory of blood demonstrated herein indicate that the PS effect does occur in the SGB of a centrifugal blood pump. The present results show that PS appeared at the PSE was approximately 90% in over the HCT_W_ range from 0 to 20%. The most conservative values, PSE_min_(dx = 25 µm), were 75% or greater, demonstrating that the PS effect occurred with this level of efficiency. In addition, at HCT_W_ values less than 20%, each PSE value was significantly higher than PSE_L_. Therefore, this study confirmed that a significant level of PS occurred at the HCT_W_ values employed. However, above this HCT_W_ range, the PSE was essentially equivalent to the PSE_L_. The cutoff HCT_W_ value for performing an evaluation would therefore be 20%.

### Limitations and future work

The use of a hydrodynamically levitated centrifugal blood pump complicates the study of the PS effect because the bearing gap is affected by the hydrodynamic force. This, in turn, is determined by the rotational speed of the pump and the viscosity of the working fluid. For future study, it is important to investigate the inhibition of hemolysis by PS. However, because the bearing gap changes with increases in the rotational speed, the extent of hemolysis is also increased as the shear stress becomes greater in the gap, and this phenomenon combines with the PS effect. In addition, although a means of estimating dx when working with blood is proposed in this study, the accuracy of this estimation affects the accuracy with which the PSE value can be obtained. Therefore, it would be desirable to stabilize the gap so that it is not affected by either the impeller rotation or the blood viscosity. It should also be noted that the extent to which the pump used in the present work induced hemolysis increased along with the rotational speed [18]. At present, inhibition of hemolysis by the PS effect is not fully understood. According to Fig. 5a, we speculate that PSE values greater than 90% might be required before this inhibition becomes significant.

As a conventional approach to reduce the hemolysis of a hydrodynamically levitated centrifugal blood pump, the researchers have tried to develop an SGB to levitate the rotor at a larger gap in order to reduce the shear stress [18]. Although the approach to decrease the bearing gap size would cause plasma skimming, the proposed method would allow ease of observation because the smaller gap size decreases PSE_L_, as shown in Eqs. (6) and (7). In the future, the relationship among the gap size, the PSE, and the hemolysis should be investigated.

With regard to theoretical limitations, the optical scattering coefficient, *µ*_s_, in Eq. (2) can be determined in the case that the RBCs are uniformly distributed over the ridge. In contrast, nonuniform distribution will reduce the value of *Q* because the RBCs may begin to overlap one another, which in turn can cause PSE to be overestimated. However, as shown by the high-speed images in Fig. 3, at HCT_W_ = 20 and 15%, there was a homogeneous dispersion of RBCs over the ridge. In addition, the PSE_L_ term in Eq. (6) is the most important limitation when evaluating the PS effect. Thus, the PSE_L_ determines whether the PSE can be evaluated, so it is important to examine these variables when assessing the PS effect in the high HCT range.

The limitation reflected in the PSE_L_ value originates from the use of two-dimensional imaging, because this type of imaging cannot provide data in the case that *Q* is larger than 1. It is possible that optical coherence tomography [25–27] might be able to overcome this limitation, because this technique can produce cross-sectional images such as that shown in Fig. 1d. Our own group has also employed a hyperspectral imaging system to monitor thrombus formation in the same pump used in this study [28–31]. This system can generate images based on the optical absorption of hemoglobin in the ridge area by acquiring data at multiple wavelengths, such that the hemoglobin concentration over the ridge can be calculated based on the Beer–Lambert law. As a way not to rely on an optical method to evaluate the PS effect, for an invisible pump, an impedance measurement method of blood may be useful because such a method can measure the local RBC density [32]. In addition, a magnetically levitated centrifugal blood pump also has potential for the measurement of blood viscosity, which corresponds to HCT_R_ [33]. Finally, it is important to note that the imaging of the flow of platelets within an SGB in a rotary blood pump has not yet been achieved. Thus, at present, it is completely unknown if platelets can escape from the ridge area in the same manner as RBCs. Since it is extremely difficult to image platelets under visible or near-infrared light without labeling, fluorescence imaging has typically been employed for this purpose [34–36]. However, the rate of blood flow inside the hydrodynamic bearing of a rotary blood pump is very high, so it will be necessary to development a process allowing the high-speed fluorescent imaging of platelets.

## Conclusion

This work established a method for the limitations associated with evaluating the PSE within an SGB in a hydrodynamically levitated centrifugal blood pump. The results of this study demonstrate that a PS effect occurs in this type of pump in human blood at or below a hematocrit of 20%. We believe that the PS effect in a rotary blood pump should be investigated in more detail to permit future advances, such as ultra-miniaturization or hemocompatibility, and the method proposed herein should assist in such research.

## Electronic supplementary material

Below is the link to the electronic supplementary material.

Supplementary file1(HCT_W_=35% in Fig.3) (MPEG 6388 kb)

Supplementary file2(HCT_W_=30% in Fig.3) (MPEG 6394 kb)

Supplementary file3(HCT_W_=25% in Fig.3) (MPEG 6394 kb)

Supplementary file4(HCT_W_=20% in Fig.3) (MPEG 6396 kb)

Supplementary file5(HCT_W_=15% in Fig.3) (MPEG 6394 kb)

Supplementary file6(HCT_W_=10% in Fig.3) (MPEG 6376 kb)
